# Long non-coding RNA Gm37494 alleviates osteoarthritis chondrocyte injury via the microRNA-181a-5p/GABRA1 axis

**DOI:** 10.1186/s13018-022-03202-5

**Published:** 2022-06-10

**Authors:** Aidong Yuan, Penghuan Wu, Zhinian Zhong, Zhengyan He, Wenhu Li

**Affiliations:** Department of Joint and Sports Medicine, The First People’s Hospital of Shaoguan City, No.3 Dongdi South Road, Zhenjiang District, Shaoguan, 512000 Guangdong People’s Republic of China

**Keywords:** Osteoarthritis, Long non-coding RNA Gm37494, microRNA-181a-5p, GABRA1, Chondrocyte, Inflammation, Damage

## Abstract

**Objective:**

This study was conducted to investigate the effect of long non-coding RNA (lncRNA) Gm37494 on osteoarthritis (OA) and its related molecular mechanism.

**Methods:**

The cartilage tissues were obtained from OA patients, and an OA mouse model was induced by the destabilization of the medial meniscus, followed by measurement of Gm37494, microRNA (miR)-181a-5p, GABRA1 mRNA, and the encoded GABA_A_R_α1_ protein expression. Thereafter, a cellular model was induced by interleukin-1β (IL-1β) treatment in chondrocytes, followed by ectopic and silencing experiments. Chondrocyte proliferation was detected by CCK-8 and EdU assays, chondrocyte apoptosis by flow cytometry and western blot, and the levels of inflammatory factors by ELISA. The binding of Gm37494 to miR-181a-5p was evaluated by dual-luciferase reporter gene and RIP assays, and that of GABRA1 to miR-181a-5p by dual-luciferase reporter gene and RNA pull-down assays.

**Results:**

OA patients and mice had decreased GABRA1 mRNA and GABA_A_R_α1_ protein levels and elevated miR-181a-5p expression in cartilage tissues. Additionally, Gm37494 was poorly expressed in OA mice. Mechanistically, Gm37494 directly bound to and inversely modulated miR-181a-5p that negatively targeted GABRA1. In IL-1β-induced chondrocytes, Gm37494 overexpression enhanced cell proliferation and suppressed cell apoptosis and inflammation, whereas further miR-181a-5p up-regulation or GABRA1 silencing abolished these trends.

**Conclusions:**

Conclusively, Gm37494 elevated GABRA1 expression by binding to miR-181a-5p, thus ameliorating OA-induced chondrocyte damage.

**Supplementary Information:**

The online version contains supplementary material available at 10.1186/s13018-022-03202-5.

## Introduction

Osteoarthritis (OA) is a relatively common degenerative disease with a still unclear etiology and pathogenesis, characterized by aggravating cartilage erosion [1]. It is widely accepted that inflammation plays a vital role in the occurrence and development of OA [2, 3]. OA poses a worldwide health burden and becomes a severe public health issue along with the aging of the population [4]. However, OA management has exhibited limited effectiveness because of the complex and poorly understood pathogenesis of OA [5].

Long non-coding RNAs (lncRNAs) are a class of nucleic acid molecules longer than 200 nucleotides lacking a significant reading frame, which participate in plentiful types of diseases [6, 7]. Increasing evidence has indicated the participation of lncRNAs in course of OA, such as PCGEM1 and CIR [8, 9]. LncRNA Gm37494 is a newly discovered molecule that substantially suppresses the expression of inflammatory factors in the repair process of spinal cord injury [10]. However, the detailed effect of Gm37494 on cartilage and its contribution to the course of OA has not been elucidated. Mounting evidence identified the involvement of lncRNAs in OA development by regulating small non-coding molecule microRNAs (miRs) [11, 12]. It has been extensively discussed that miRs assume a role in musculoskeletal injuries, including tendon injuries and OA [13, 14]. Intriguingly, miR-181a-5p is a kind of miR, exerting its biological effects in a large number of diseases [15–17]. Some studies on the expression and function of miR-181a-5p in OA chondrocytes have been conducted and identified miR-181a-5p as a promoting factor in OA [18, 19].

To gain further insight into the mechanism of miR-181a-5p in OA progression, we screened for possible targets of miR-181a-5p with the bioinformatics method and predicted the targeting relationship between miR-181a-5p and gamma-aminobutyric acid type A receptor subunit alpha1 (GABRA1), a subunit of GABA receptors. It was reported that GABA receptors possessed regulatory functions in inflammation or the immune response [20, 21]. Up to date, there is no research exploring the relationship between GABRA1 and OA. The present study predicted the binding among Gm37494, miR-181a-5p, and GABRA1. On this basis, a hypothesis was proposed that Gm37494 might affect OA development by up-regulating GABRA1 via miR-181a-5p. The purpose of this research was to ascertain the function of Gm37494 in OA and the downstream mechanisms.

## Materials and methods

### Clinical samples

The cartilage tissues were collected from 20 OA patients undergoing total knee replacement in the First People’s Hospital of Shaoguan City. The normal cartilage tissues were harvested from 20 donors after they died, who had no OA history. The information of the subjects is listed in Table 1. Cartilage tissues were obtained from the transition area of the femur and the weight-bearing area of the lateral malleolus. The OA was diagnosed based on the diagnostic criteria proposed by the American College of Rheumatology [22]. The influence of any factors that might affect the condition of OA was excluded. All participants or their family members had acquainted with the objective and requirements of the experiments before signing the written informed consent. This study was conducted with the approval of the ethical committee of the First People’s Hospital of Shaoguan City and by conforming to the *Declaration of Helsinki*.

**Table 1:** Clinical information of subjects

Clinical information	Control group	OA group
Gender		
Male (cases)	12	14
Female (cases)	8	6
Age		
≤ 60 (cases)	13	11
> 60 (cases)	7	9
Mean age (years)	58.35 ± 7.45	56.25 ± 9.25
Body mass index	25.55 ± 1.82	21.83 ± 1.37

### A mouse model of OA

The model of traumatic knee OA was established with the destabilization of the medial meniscus (DMM) method. Concretely, male C57BL/6J mice (8 weeks old) were obtained from Henan Experimental Animal Center. The mice were anesthetized with 30 mg/kg pentobarbital sodium. In the DMM group (*n* = 10), after the exposure of the right knee joint through a medial capsule incision, the extensor muscle was gently flexed, and then the medial meniscus was transected. Finally, the medial capsule and skin were sutured. In the sham group (*n* = 10), the same operation was performed, apart from the medial meniscus ligament dissection. All procedures were ratified by the Ethical Committee for Animal Research of the First People’s Hospital of Shaoguan City, and mice were treated in accordance with national guidelines for the nursing and usage of laboratory animals.

### Safranin O-fast green staining

Four weeks after the DMM operation, the joint tissue sections of the right knees were dewaxed, followed by gradient ethanol hydration (anhydrous, 95%, 85%, and 70% ethanol, with 5 min each). After three washes in phosphate-buffered saline (PBS), the sections were stained with hematoxylin staining solution for 3–5 min, washed fully in running water, and stained with 0.02% fast green solution for 3 min. Thereafter, the sections were washed with 1% glacial acetic acid and stained with 0.1% safranin O for 3 min. The sections were washed using 95% ethanol, dehydrated using anhydrous ethanol, and cleared using xylene. The sections were rapidly added with neutral gum dropwise and sealed with coverslips, after which, staining was observed using a microscopy. Next, the Osteoarthritis Research Society International (OARSI) standard [23] was applied for histological scoring based on the following four criteria: matrix staining, cartilage structure, chondrocyte clustering, and tideline integrity with the total score of 24. The higher the score, the more severe the degeneration and destruction of the articular cartilage.

### Chondrocyte isolation, culture, and transfection

Chondrocytes were extracted from OA cartilage tissues with reference to the description of previous literature [24]. Specifically, the mice were euthanized four weeks after the DMM operation. Cartilage tissues were harvested and cut into small pieces in a sterile environment. Trypsin (Sigma-Aldrich, St. Louis, MO, USA) was employed to digest the cartilage tissues. After growing to a monolayer, chondrocytes were cultured in Dulbecco's modified eagle medium (Gibco, Carlsbad, California, USA) with 10% fetal bovine serum (FBS, Gibco) and 100 U/mL penicillin. After that, chondrocytes were seeded onto plates and 100 mg/mL streptomycin (Gibco) was added at 37 °C. Adherent chondrocytes with a confluence of 70–80% were cultured in a serum-free medium for 12 h and induced using 10 ng/mL interleukin-1β (IL-1β) for 2 h to simulate the pathological changes of OA. HEK 293 T cells (American Type Culture Collection, Manassas, VA, USA) were cultured in a Minimum Essential Medium (Gibco) mixed with 10% FBS. Overexpression (oe)-Gm37494 vectors, oe-negative control (NC), miR-181a-5p mimic, mimic NC, miR-181a-5p inhibitor, inhibitor NC, short hairpin RNA (sh)-GABRA1 vectors, and sh-NC vectors were purchased from Hanbio Biotechnology Co., Ltd. (Shanghai, China). Transfection was performed using Lipofectamine 2000 reagents (Invitrogen, Carlsbad, CA, USA) as per the instructions of the manufacturer. Subsequent experiments were performed 48 h after transfection.

### Cell Counting Kit-8 (CCK-8) assay

After transfection, cells were seeded on 96-well plates (2 × 10^3^ cells/well) with 3 replicates for each sample, and each well was added with 10 μL CCK-8 solution. After incubation in CO_2_ incubators for 0 h, 24 h, 48 h, and 72 h, respectively, the optical density at 450 nm was tested with a microplate reader (Bio-Rad 680; Bio-Rad, Hercules, CA, USA).

### 5-Ethynyl-2′-deoxyuridine (EdU) assay

The BeyoClick™ EdU-555 Cell Proliferation Assay Kit (Beyotime, Shanghai, China; C0075L) was adopted to detect cell proliferation. The 2 × EdU working solution pre-warmed at 37 °C was added to the cells in 96-well plates (5 × 10^3^ cells/well) in equal volume with the original culture medium, that is, the final concentration of EdU was 10 µM. Thereafter, the plates were incubated in the incubator for 4 h. After the removal of the culture medium, cells were incubated for 15 min using 1 mL of 4% paraformaldehyde fixative at room temperature. After the discarding of the fixative, the cells in each well were washed using 1 mL washing solution 3 times, with each time for 3–5 min. Afterward, the wash solution was discarded, and the cells in each well were cultured at room temperature with 1 mL permeabilization solution made of PBS with 0.3% Triton X-100 for 10–15 min. After the permeabilization solution was removed, the cells in each well were washed twice with 1 mL wash solution for 5 min each time. The Click Additive was dissolved in deionized water, mix completely, and stored at − 20 °C. The Click Reaction Solution (0.5 mL) was added to each well after the discarding of the washing solution in the previous step to make the reaction solution cover the samples uniformly. After 30 min of cell incubation at room temperature in the dark, the Click reaction solution was aspirated and cells were washed with the washing solution 3 times for 5 min each time. PBS was applied to dilute 4′,6-diamidino-2-phenylindole (DAPI; 1000 × ). After dilution, each well was added with 1 mL DAPI for nuclear staining. Then, 1 × DAPI solution was aspirated, and cells were washed 3 times with washing solution for 5 min each time, followed by fluorescence detection. Three fields of view were randomly selected under 200 × magnifications, where EdU-stained cells (proliferating cells) and DAPI-stained cells (total cells) were counted. Cell proliferation rate = the number of proliferating cells/the total number of cells × 100%. The experiment was repeated 3 times.

### Enzyme-linked immunosorbent assay (ELISA)

The levels of inflammatory factors, including tumor necrosis factor-α (TNF-α), interleukin (IL)-6, and IL-10, were measured using corresponding ELISA kits (R&D Systems, Minneapolis, MN, USA) as per the procedure provided by the kits. Specifically, the samples after treatment were added to an ELISA plate for overnight coating at room temperature. The cell culture medium was carefully discarded and cells were washed 3 times with PBS (5 min each time). The plate were blocked with 5% bovine serum albumin (BSA) blocking solution (100 μL per well) for 1 h. The corresponding primary antibodies were diluted with PBS (containing 5% BSA) and added to the 96-well plate (100 μL per well) for 3-h binding. The plate was washed 3 times with PBS for 5 min each time. Thereafter, the horseradish peroxidase (HRP)-conjugated secondary antibody was washed with PBS (containing 5% BSA) and added to the 96-well plate for 1-h binding. After that, the plate was washed 3 times with PBS for 5 min each time. Substrate (10 μL) was added to the plate and placed at 37 °C for 10–15 min, followed by the determination of the absorbance values at 450 nm on the microplate reader (Bio-Rad 680; Bio-Rad). The experiment was repeated 3 times.

### Flow cytometry

Apoptosis was measured using an Annexin V-fluorescein isothiocyanate (FITC) Kit (Beyotime). Cells were trypsinized, collected by centrifugation, and resuspended using the binding buffer. After 15 min of incubation with Annexin V-FITC at room temperature, a flow cytometer was applied to detect the apoptosis rate. Apoptosis results were determined as described below. Briefly, horizontal axis stood for Annexin V intensity and vertical axis indicated PI staining. Mechanically damaged cells were located in the upper left quadrant. Late apoptotic or necrotic cells were located in the upper right quadrant. Negative normal cells were located in the lower left quadrant. Early apoptotic cells were located in the lower right quadrant. All Annexin V^+^ cells were defined as apoptotic cells in the analysis. All experiments were repeated 3 times.

### Western blot

Cells or tissues were lysed with Radio Immunoprecipitation Assay lysis buffer (Beyotime) on ice for 15 min and centrifuged at 13,000*g* for 5 min. Then, the total protein concentration was measured with the bicinchoninic acid kit (Beyotime). After being added to the loading buffer, the protein was denatured in boiling water for 10 min. The protein underwent electrophoresis firstly at 80 V for 30 min and at 120 V for 90 min after bromophenol blue entered the separation gel. Subsequently, the protein was electroblotted to polyvinylidene fluoride membranes at 250 mA in an ice bath for 100 min. The membranes were washed 3 times with the washing solution for 1–2 min per time and blocked in sealing solution for 2 h. Thereafter, the membranes underwent overnight incubation with primary antibodies (Abcam, Cambridge, UK; 1:1000) against γ-aminobutyric acid A receptor α1 subunit (GABA_A_R_α1_, ab252430), B-cell lymphoma-2 (Bcl-2)-Associated X (Bax, ab32503), cleaved caspase 3 (ab32042), Bcl-2 (ab32124), glyceraldehyde-3-phosphate dehydrogenase (GAPDH, ab9485) at 4 °C, followed by 3 washes with Tris-buffered saline with Tween 20 (TBST) for 10 min each time. Afterward, the membranes were cultured for 2 h with HRP-conjugated goat anti-rabbit Immunoglobulin G (IgG) secondary antibodies (Beyotime; A0208, 1:1000) at room temperature and washed as the above procedure. Blots were detected with electrochemiluminescence (P0018FS, Beyotime) on a chemiluminescence imaging system (Bio-Rad). Result analysis was conducted using Quantity One v4.6.2 software, and the relative protein levels were expressed by the ratio of the grayscale value of the corresponding protein bands to the GAPDH protein band. The experiments were repeated three times, and the mean value was calculated.

### Quantitative real-time polymerase chain reaction (qRT-PCR)

Total RNA was isolated from cartilage tissues and chondrocytes with TRIZOL (Invitrogen, Carlsbad, CA, USA). The concentration of the extracted RNA was measured on a DU-640 spectrometer (Beckman, San Jose, CA, USA). Afterward, the cDNA synthesis kit (Promega, Madison, WI, USA) was applied to synthesize the first strand of cDNA, and all operations were performed as per the manuals of the kit. Real-time PCR was carried out with SYBR Green Mix (TaKaRa, Tokyo, Japan) on a Biosystems 7300 qRT-PCR system (ABI, Foster City, CA, USA), with three replicates for each reaction. The data were calculated based on the 2^−ΔΔCt^ method with GAPDH or U6 as the internal reference: ΔΔCt = the experimental group (Ct target gene—Ct internal reference)—the control group (Ct target gene—Ct internal reference). Primers are available in Table 2.

**Table 2:** Primer sequences for qRT-PCR

Targets	Sequences (5′–3′)
miR-181a-5p-F-hsa	CCGCGAACATTCAACGCTGTCG
miR-181a-5p-R-hsa	ATCCAGTGCAGGGTCCGAGG
U6-F-F-hsa	CAAATTCGTGAAGCGTTCCATAT
U6-R-F-hsa	GCTTCACGAATTTGCGTGTCATCCTTGC
miR-181a-5p-F-mus	GGGAACATTCAACGCTGTCG
miR-181a-5p-R-mus	GTGCGTGTCGTGGAGTCG
U6-F-F-mus	GCTTCGGCAGCACATATACTAAAAT
U6-F-R-mus	CGCTTCACGAATTTGCGTGTCAT
Gm37494-F-mus	GGAGATTCCTAAGAA
Gm37494-R-mus	GACTTTGTTGCTGTA
GABRA1-R-hsa	ATGCGGATTTCGTCCTGACT
GABRA1-R-hsa	GCCTCGAGCTCCATCATTCT
GABRA1-F-mus	GAGGGTATGCGTGGGATG
GABRA1-R-mus	GCTTGACTTCTTTCGGTTCTAT
GAPDH-F-mus	CCCTTAAGAGGGATGCTGCC
GAPDH-R-mus	ACTGTGCCGTTGAATTTGCC
GAPDH-F-hsa	AATGGGCAGCCGTTAGGAAA
GAPDH-R-hsa	GCGCCCAATACGACCAAATC

### Dual-luciferase reporter gene assay

Possible binding sites of miR-181a-5p to Gm37494 were obtained through starBase (http://starbase.sysu.edu.cn/) and those of miR-181a-5p to GABRA1 were predicted by TargetScan (http://www.targetscan.org/vert_72/). After being designed and synthesized based on the predicted results, the wild-type and mutant sequences (wt-Gm37494, mut-Gm37494, wt-GABRA1, and mut-GABRA1) were inserted into the luciferase reporter vector (PGL3-Promotor) and co-transfected into HEK 293T cells with miR-181a-5p mimic (30 nM) or mimic NC. Firefly and Renilla luciferase activities of cells were measured using a dual-luciferase reporter kit (Promega) and photometer (Turner BioSystems, Sunnyvale, CA, USA). With Renilla luciferase activity as an internal control, the calculated ratio of firefly luciferase activity to Renilla luciferase activity represented the relative activity of luciferase.

### RNA pull-down assay

RNA pull-down assays were performed using the Pierce™ Magnetic RNA–Protein Pull-Down Kit (Millipore, Billerica, MA, USA). Biotinylated miR-181a-5p probes or NC (Geneseed, Guangzhou, China) were incubated with cell lysates for 2 h at 25 °C. The complexes were captured with streptavidin-labeled immunomagnetic beads at 25 °C for 1 h and then incubated with proteinase K-containing buffer at 25 °C for 1 h. The eluted complexes were determined by qRT-PCR.

### RNA binding protein immunoprecipitation (RIP) assay

Cells were washed twice with pre-chilled PBS, centrifuged at 1500 rpm for 5 min, and fully titrated and mixed with an equal volume of RIP lysis. The magnetic beads were resuspended using 100 μL RIP Wash Buffer and added with 5 μg Argonaute 2 (Ago2) antibodies (ab186733, 1:100, Abcam) and IgG antibodies (the NC, ab172730, 1:100, Abcam), followed by 30 min of incubation at room temperature. The centrifuge tubes were placed on a magnetic stand with the supernatant discarded, added with 500 μL RIP Wash Buffer, vortexed, and shaken, with the removal of the supernatant, which was repeated once. The tubes were added with 500 μL RIP Wash Buffer, vortexed, shaken, and placed on ice. The prepared bead tubes were placed on the magnetic stand with the discarding of the supernatant, and each tube was added with 900 μL RIP Immunoprecipitation Buffer. The previously prepared cell lysate was quickly thawed and centrifuged at 14,000 rpm and 4 °C for 10 min. Afterward, 100 μL supernatants were added to the magnetic bead-antibody complex, followed by overnight incubation at 4 °C and transient centrifugation. The tubes were placed on the magnetic stand, and the supernatant was removed. Each tube was added with 500 μL RIP Wash Buffer, vortexed, and shaken. Thereafter, the tubes were placed on the magnetic stand with the supernatant removed. After the tubes were washed 6 times, each sample was added with 150 μL Proteinase K Buffer to resuspend the magnetic bead-antibody complex, followed by 30-min incubation at 55 °C. The tubes were placed on the magnetic stand and the supernatant was harvested. Gene expression was detected by qRT-PCR after RNA extraction. Each experiment was repeated 3 times.

### Statistical analysis

Data were processed using GraphPad Prism 7.0 (GraphPad Software, San Diego, CA, USA) and presented as mean ± standard deviation. Data analysis was performed using the *T* test when the two groups were compared and one-way analysis of variance when multiple groups were compared. Tukey’s test was utilized for post hoc multiple comparisons. A statistical significance was defined as *P* < 0.05.

## Results

### Gm37494 expression was down-regulated in OA mice

**Fig. 1:**
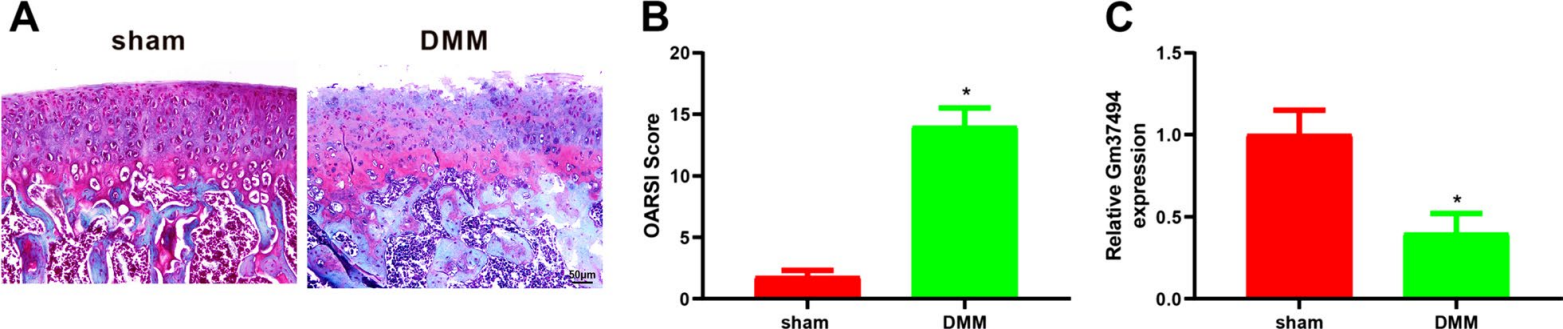
Gm37494 expression was poor in a mouse model of OA. **A** the pathological changes of the knee joint of mice in the sham group and the DMM group observed by safranin O-fast green staining; **B** the OARSI score of mice in the sham group and the DMM group; **C** the expression of Gm37494 in the OA mouse model detected by qRT-PCR. *P < 0.05, compared with the sham group. *N* = 10 mice/group

First, the OA mouse model was established to clarify the relationship between Gm37494 and OA. Then, the OA mouse model was validated using safranin O-fast green staining and OARSI score. It was observed in the result of safranin O-fast green staining that versus the sham group, there were irregular cracks on the cartilage surface of the right knee joint from mice, and chondrocytes were disordered and decreased in number in the DMM group (Fig. 1A). In addition, the OARSI score of the DMM group was significantly higher than that of the sham group (Fig. 1B, *P < 0.05). These findings illustrated the successful induction of the OA mouse model. Subsequently, Gm37494 expression was assessed in the OA mouse model to initially identify the function of Gm37494 in OA. qRT-PCR results manifested that Gm37494 expression was obviously poorer in the DMM group than in the sham group (Fig. 1C, *P < 0.05), suggesting that Gm37494 was closely associated with OA.

### Gm37494 overexpression repressed IL-1β-induced chondrocyte damage

**Fig. 2:**
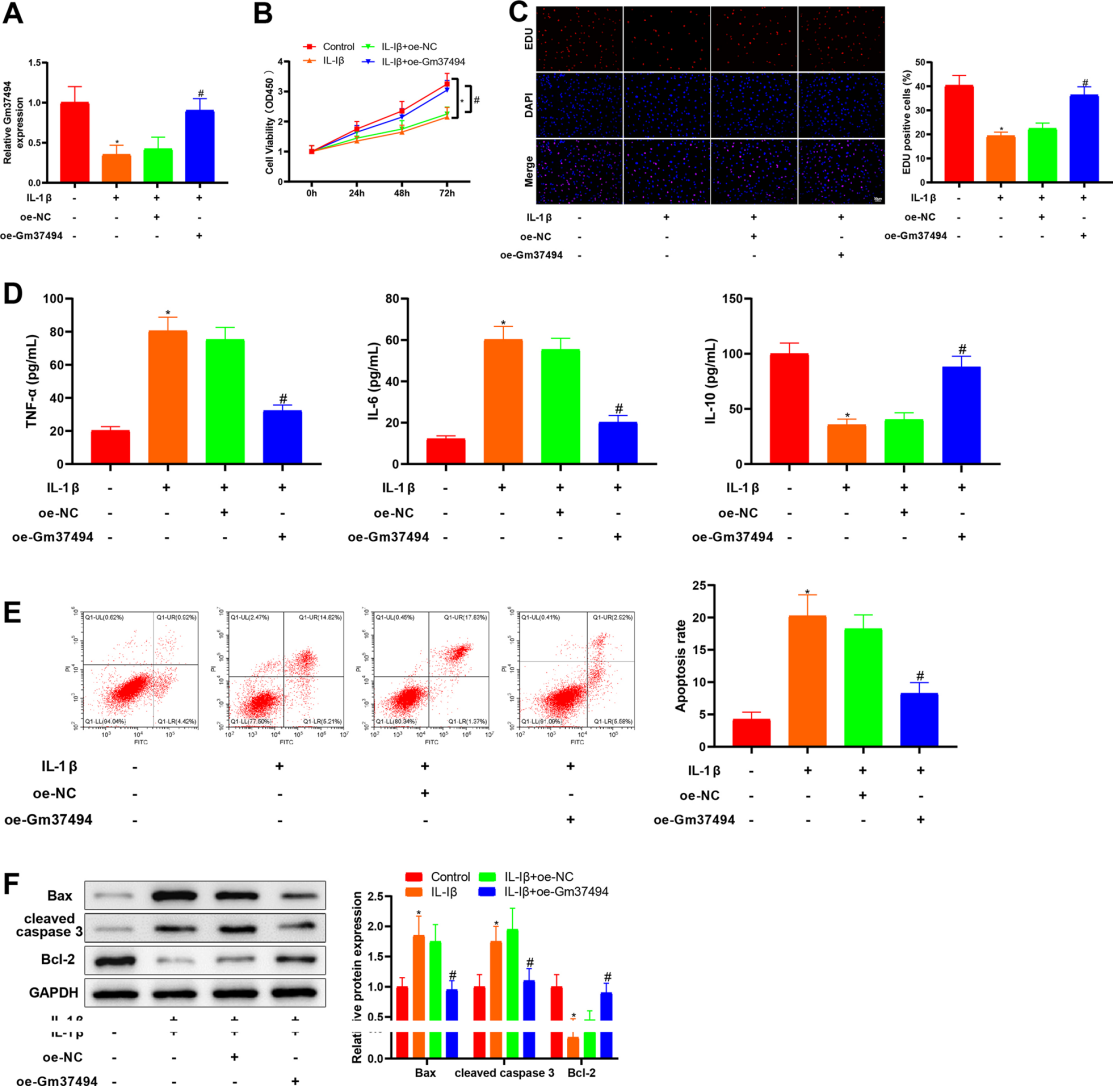
Overexpression of Gm37494 in vitro remarkably inhibits chondrocyte damage induced by IL-1β. IL-1β-induced chondrocytes were transfected with oe-Gm37494. **A** Gm37494 expression measured by qRT-PCR in IL-1β-induced chondrocytes; **B**–**C** CCK-8 (**B**) and EdU (**C**) assays to detect chondrocyte proliferation; **D** ELISA to detect the levels of inflammatory factors TNF-α, IL-6, and IL-10 in IL-1β-induced chondrocytes; **E** flow cytometry to examine chondrocyte apoptosis; **F** western blot to test the expression of apoptotic proteins Bax, Bcl-2, and cleaved caspase 3 in IL-1β-induced chondrocytes. Data were expressed as mean ± standard deviation, and each experiment was repeated 3 times. *P < 0.05, compared with the control group; ^#^*P* < 0.05, compared with the IL-Iβ + oe-NC group

The aforesaid data illustrated Gm37494 down-regulation in OA. In order to further ascertain the role of Gm37494 in OA, the Gm37494 overexpression vector, oe-Gm37494, was transfected into IL-1β-induced chondrocytes, followed by the determination of Gm37494 expression. The data revealed that Gm37494 expression was substantially decreased in the IL-1β group compared to the control group (*P < 0.05) and that significantly elevated Gm37494 expression was observed in the IL-1β + oe-Gm37494 group relative to the IL-1β + oe-NC group (Fig. 2A, ^#^*P* < 0.05). Next, we dissected the effects of Gm37494 on chondrocyte damage. With respect to the results of CCK-8 and EdU assays, IL-1β treatment remarkably diminished chondrocyte proliferation in contrast to control treatment (Fig. 2B, C, *P < 0.05). ELISA showed that IL-1β treatment evidently enhanced TNF-α and IL-6 levels and decreased IL-10 levels compared to control treatment (Fig. 2D, *P < 0.05). Flow cytometry exhibited that chondrocyte apoptosis was conspicuously elevated by IL-1β induction (Fig. 2E, *P < 0.05). Meanwhile, western blot results depicted significant increases in the expression of apoptotic proteins Bax and cleaved caspase 3 and marked declines in the anti-apoptotic protein, Bcl-2, expression in chondrocytes after IL-1β treatment versus control chondrocytes (Fig. 2F, *P < 0.05). Transfection of oe-Gm37494 into 1L-1β-treated chondrocytes alleviated the damaging effect of IL-Iβ treatment on chondrocytes and attenuated chondrocyte damage (Fig. 2B–F, ^#^*P* < 0.05). In conclusion, overexpression of Gm37494 led to evident suppression of chondrocyte damage induced by IL-1β.

### Gm37494 directly bound and negatively correlated to miR-181a-5p

**Fig. 3:**
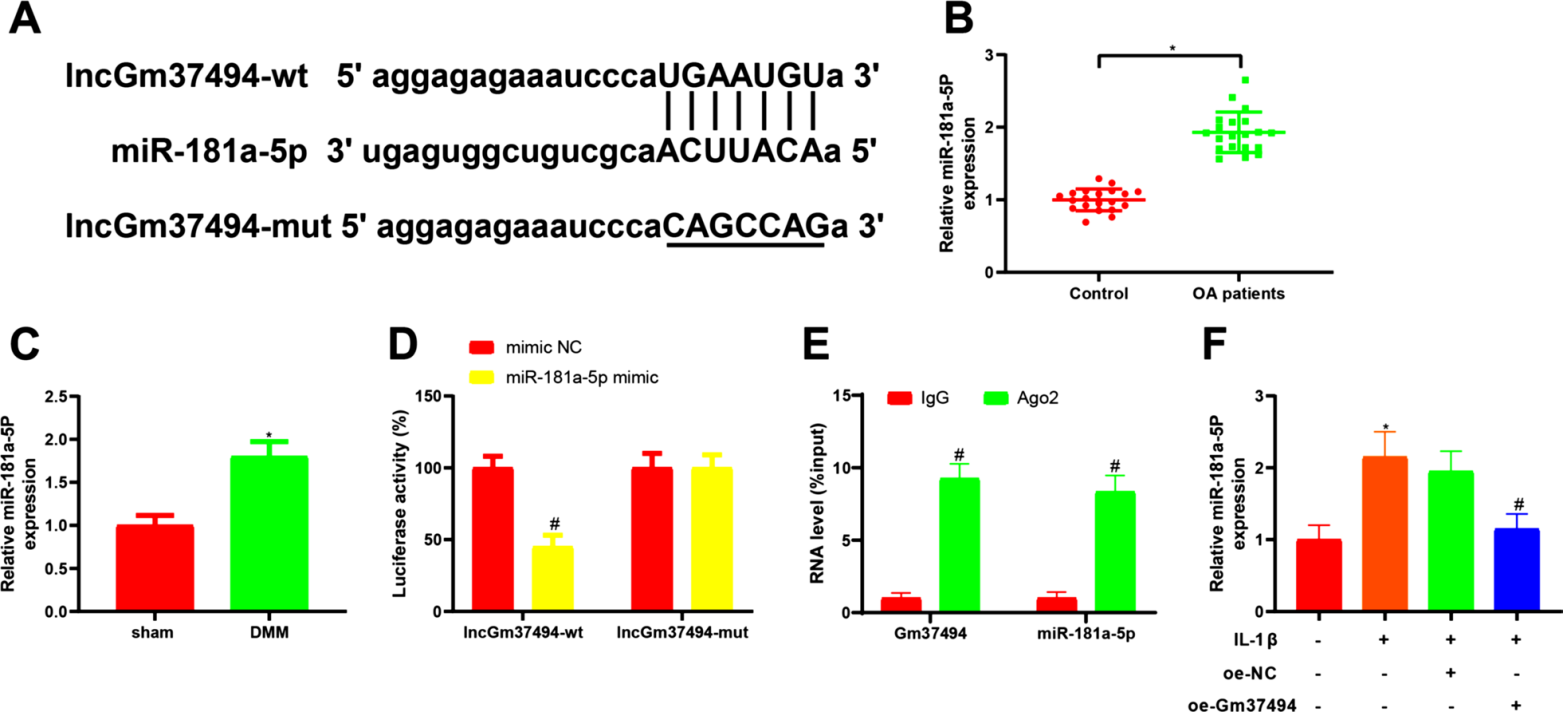
Gm37494 directly binds to and is inversely correlated with miR-181a-5p. **A** the binding site and mutant sequence between miR-181a-5p and Gm37494; **B**–**C** the expression of miR-181a-5p in patients with OA (**B**) and the mouse model of OA (**C**) determined by qRT-PCR. **D**–**E** the binding between miR-181a-5p and Gm37494 assessed by dual-luciferase reporter gene (**D**) and RIP assays (**E**); **F** the relative expression of miR-181a-5p observed by qRT-PCR after transfection of oe-Gm37494 in chondrocytes induced by IL-1β. Data were expressed as mean ± standard deviation. *N* = 20 for clinical experiments, and *N* = 10 mice/group for animal experiments. Cell experiments were repeated 3 times. *P < 0.05, compared with the control or sham group; ^#^*P* < 0.05, compared with the mimic NC, IgG, or IL-1β + oe-NC group

In the aforementioned experiments, it was found that overexpression of Gm37494 noticeably inhibited IL-1β-induced chondrocyte damage, with unclear related molecular mechanisms. It has been manifested that miR-181a-5p was tightly associated with OA [18]. Moreover, the online software starBase predicted the presence of binding sites between Gm37494 and miR-181a-5p (Fig. 3A). In this context, we speculated that the effect of in vitro overexpression of Gm37494 on 1L-1β-induced chondrocyte damage might be mediated through miR-181a-5p. Consequently, miR-181a-5p expression was measured in the tissues of OA patients and mice. The findings demonstrated considerably higher miR-181a-5p expression in cartilage tissues of OA patients or DMM mice than in normal tissues or sham-operated mice (Fig. 3B, C, *P < 0.05). Next, in order to verify our speculation, the dual-luciferase reporter gene assay was conducted, exhibiting that the luciferase activity of wt-Gm37494 was notably diminished in the miR-181a-5p mimic group in comparison with the mimic NC group (Fig. 3D, ^#^*P* < 0.05). As further reflected by RIP assay results, Gm37494 and miR-181a-5p bound by Ago2 were prominently increased versus IgG (Fig. 3E, ^#^*P* < 0.05), further suggesting that Gm37494 bound to miR-181a-5p. Afterward, miR-181a-5p expression was determined in IL-1β-treated chondrocytes after oe-Gm37494 transfection. The results of qRT-PCR documented that miR-181a-5p was prominently reduced in IL-1β-treated chondrocytes by overexpressing Gm37494 (Fig. 3F, ^#^*P* < 0.05). The above data illustrated that Gm37494 bound to miR-181a-5p and was inversely correlated with miR-181a-5p in IL-1β-induced chondrocytes.

### Up-regulation of miR-181a-5p reversed the mitigating effect of Gm37494 overexpression on IL-1β-induced chondrocyte damage

**Fig. 4:**
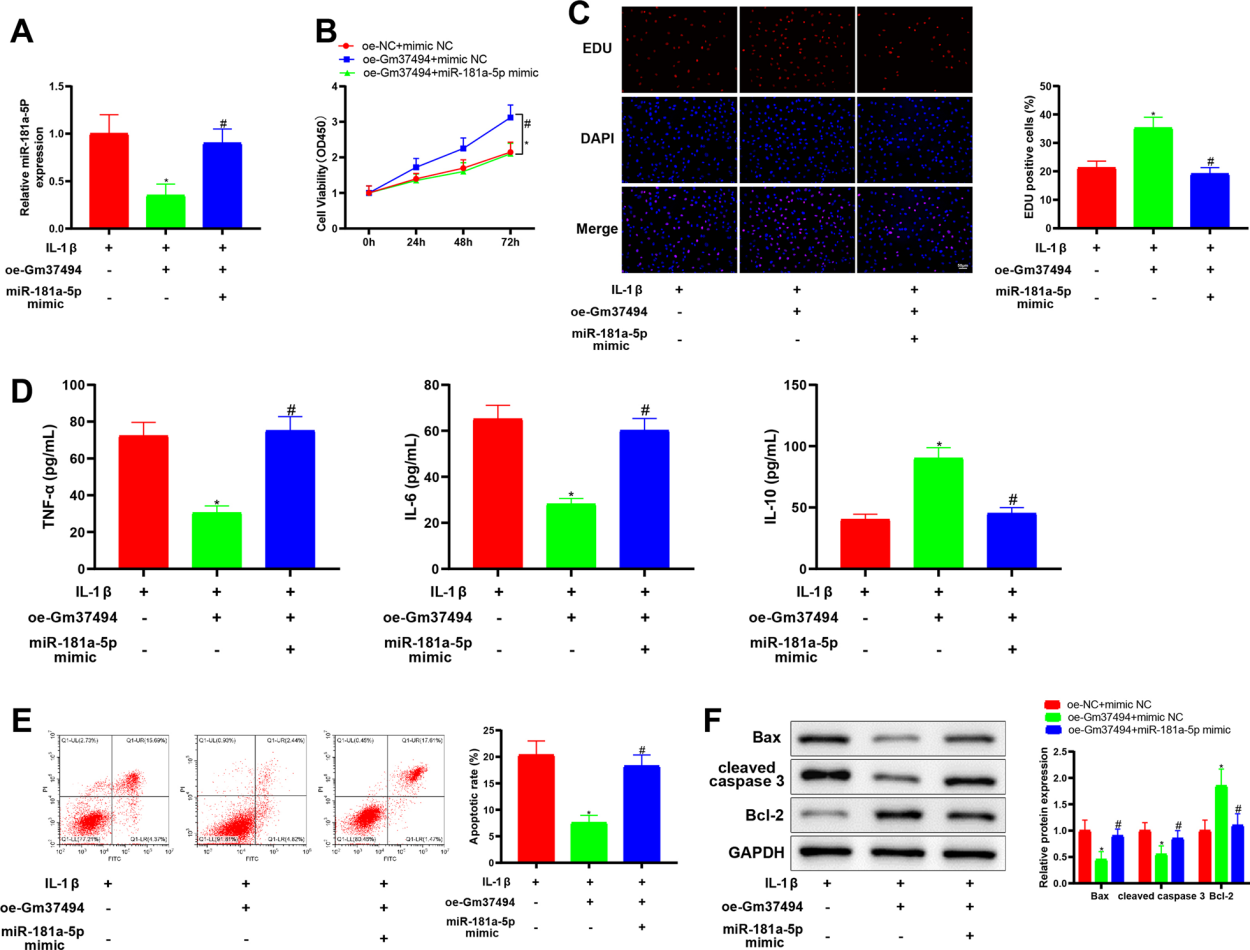
Overexpressing miR-181a-5p counteracts the attenuating influence of Gm37494 up-regulation on IL-1β-induced chondrocyte damage. Oe-Gm37494 and miR-181a-5p mimic were simultaneously transfected into 1L-1β-treated chondrocytes. **A** the expression of miR-181a-5p in 1L-1β-treated chondrocytes measured by qRT-PCR; **B**–**C** chondrocyte proliferation determined by CCK-8 (**B**) and EdU (**C**) assays; **D** the levels of the inflammatory factors TNF-α, IL-6, and IL-10 in 1L-1β-treated chondrocytes evaluated by ELISA; **E** chondrocyte apoptosis tested by flow cytometry; **F** the expression of apoptotic proteins Bax, Bcl-2, and cleaved caspase 3 in 1L-1β-treated chondrocytes detected by western blot. Data were expressed as mean ± standard deviation, and each experiment was repeated 3 times. *P < 0.05, compared with the oe-NC + mimic NC group; ^#^*P* < 0.05, compared with the oe-Gm37494 + mimic NC group

From the aforesaid experiments, it was observed that Gm37494 directly bound to and was negatively correlated with miR-181a-5p. To further clarify whether Gm37494 ameliorated chondrocyte damage through miR-181a-5p in OA, oe-Gm37494 and miR-181a-5p mimic were simultaneously transfected into IL-1β-treated chondrocytes. qRT-PCR to assess the transfection efficiency displayed that in contrast to the oe-Gm37494 + mimic NC group, miR-181a-5p expression was signally increased in the oe-Gm37494 + miR-181a-5p mimic group (Fig. 4A, ^#^*P* < 0.05). Subsequently, we evaluated the effects of Gm37494-modulating miR-181a-5p on chondrocyte damage. miR-181a-5p mimic negated the alleviatory effect of oe-Gm37494 alone on chondrocyte damage and exacerbated chondrocyte damage. Specifically, CCK-8 and EdU assays demonstrated that in contrast to the oe-Gm37494 + mimic NC group, chondrocyte proliferation was appreciably reduced in the oe-Gm37494 + miR-181a-5p mimic group (Fig. 4B, C, ^#^*P* < 0.05). In addition, it was noted from ELISA data that relative to the oe-Gm37494 + mimic NC group, IL-10 levels were substantially diminished but TNF-α and IL-6 levels were considerably enhanced in the oe-Gm37494 + miR-181a-5p mimic group (Fig. 4D, ^#^*P* < 0.05). Meanwhile, the data of flow cytometry and western blot revealed noticeable elevations in chondrocyte apoptosis and Bax and cleaved caspase 3 levels and conspicuous declines in Bcl-2 levels (Fig. 4E–F, ^#^*P* < 0.05). The aforementioned results suggested that Gm37494 overexpression decreased miR-181a-5p expression to repress IL-1β-induced chondrocyte damage.

### GABRA1 was a possible target of miR-181a-5p in IL-1β-induced chondrocytes

**Fig. 5:**
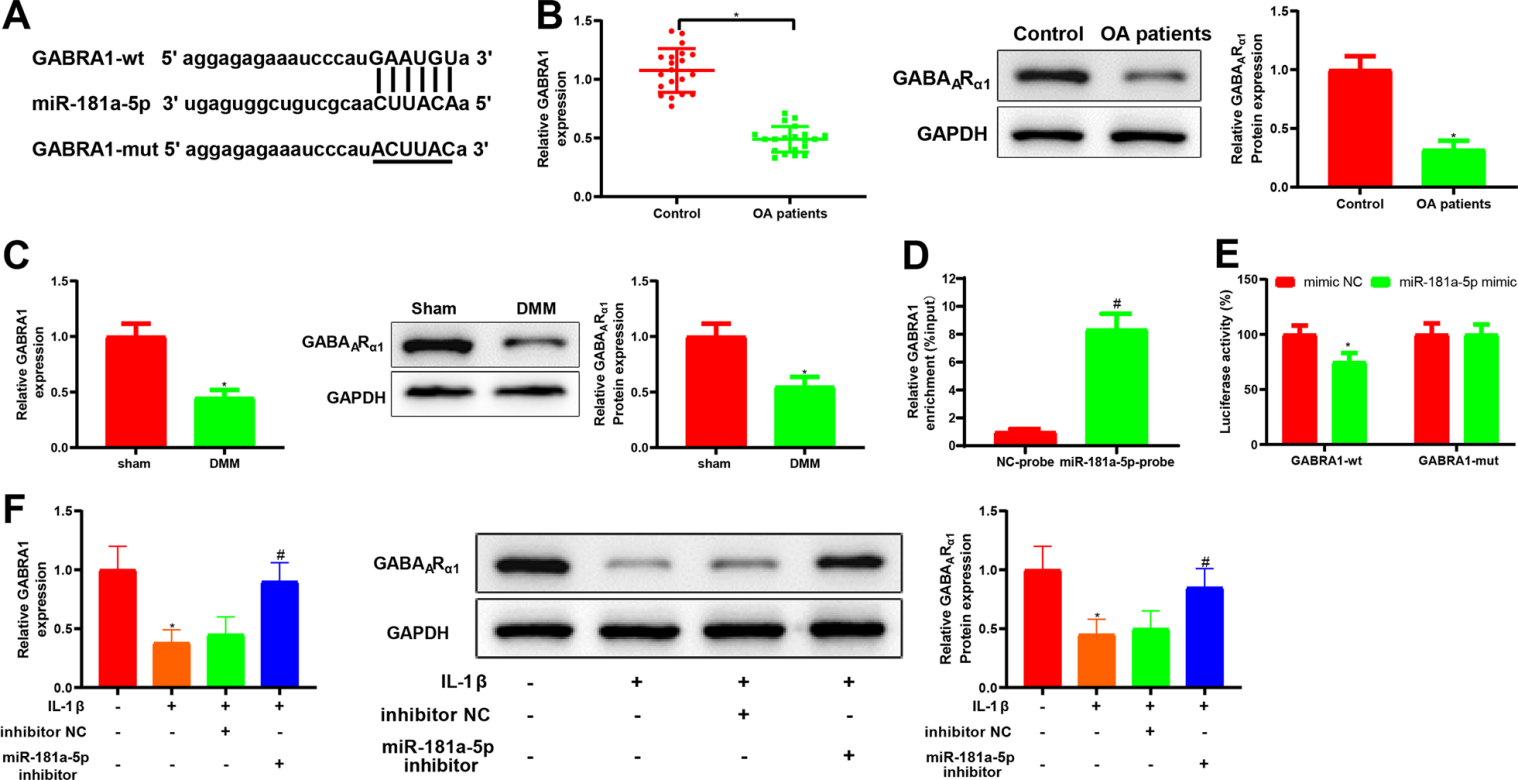
GABRA1 is targeted by miR-181a-5p. **A** the binding site and mutation sequence between miR-181a-5p and GABRA1; **B**–**C** qRT-PCR and western blot to detect the levels of GABRA1 mRNA and the encoded protein GABA_A_R_α1_, respectively, in patients with OA (**B**) and the mouse model of OA (**C**); **D**–**E** the binding between miR-181a-5p and GABRA1 tested by RNA pull-down (**D**) and dual-luciferase reporter gene (**E**) assays; **F** the levels of GABRA1 mRNA and the encoded protein GABA_A_R_α1_ assessed by qRT-PCR and western blot, respectively, after transfection of miR-181a-5p inhibitor in chondrocytes induced by IL-1β. Data were expressed as mean ± standard deviation. *N* = 20 for clinical experiments, and *N* = 10 mice/group for animal experiments. Cell experiments were repeated 3 times. *P < 0.05, compared with control, sham, or mimic NC group; ^#^*P* < 0.05, compared with the NC-probe or 1L-1β + inhibitor NC group

The above experiments elaborated that Gm37494 suppressed OA progression by down-regulating miR-181a-5p, but the specific downstream target protein was enigmatic. Thereafter, the online bioinformatics tool TargetScan was used to search endogenous target genes of miR-181a-5p, which predicted that miR-181a-5p had a putative binding site to GABRA1 (Fig. 5A). We therefore conjectured that Gm37494-mediating miR-181a-5p expression might orchestrate chondrocyte injury by altering the downstream protein GABRA1. Next, GABRA1 mRNA levels and the encoded protein GABA_A_R_α1_ expression were detected in tissues from OA patients and OA mice. The data demonstrated that GABRA1 mRNA levels and the encoded protein GABA_A_R_α1_ expression were obviously lower in the cartilage tissues of OA patients or DMM mice than in normal tissues or sham-operated mice (Fig. 5B, C, *P < 0.05). Afterward, to confirm our conjecture, we performed RNA pull-down and dual-luciferase reporter gene assays. The RNA pull-down assay manifested that GABRA1 was captured by the biotinylated miR-181a-5p probe (Fig. 5D, ^#^*P* < 0.05). The results of the dual-luciferase reporter gene assay documented that the relative luciferase activity of wt-GABRA1 decreased obviously after miR-181a-5p mimic transfection (Fig. 5E, *P < 0.05), further indicating that GABRA1 was a potential target of miR-181a-5p. miR-181a-5p inhibitor was transfected into IL-1β-induced chondrocytes to detect GABRA1 mRNA and GABA_A_R_α1_ protein levels. In detail, as discovered in qRT-PCR results, GABRA1 mRNA levels were distinctly augmented in the IL-1β + miR-181a-5p inhibitor group versus the IL-1β + inhibitor NC group (^#^*P* < 0.05). Likewise, western blot results also exhibited conspicuous elevations in GABA_A_R_α1_ protein levels of the IL-1β + miR-181a-5p inhibitor group in comparison to the IL-1β + inhibitor NC group (Fig. 5F, ^#^*P* < 0.05). These results suggested that miR-181a-5p targeted and inversely modulated GABRA1 expression in IL-1β-induced chondrocytes.

### GABRA1 down-regulation nullified the relieving influence of Gm37494 overexpression on IL-1β-induced chondrocyte damage

**Fig. 6:**
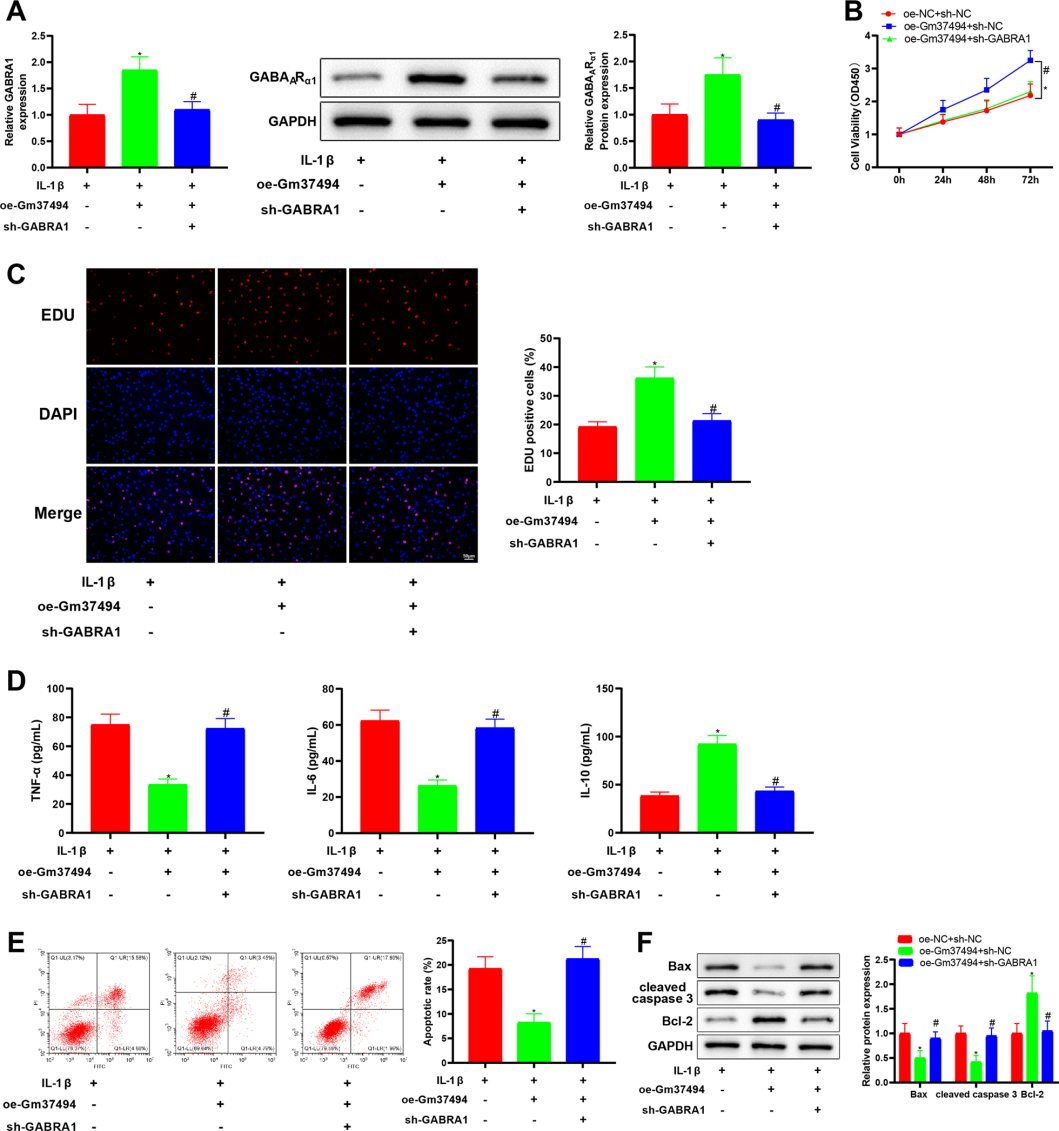
Gm37494 relieves IL-1β-induced chondrocyte damage by increasing GABRA1 expression. Oe-Gm37494 and sh-GABRA1 were simultaneously transfected into 1L-1β-treated chondrocytes. **A** qRT-PCR and western blot detection of the levels of GABRA1 mRNA and the encoded protein GABA_A_R_α1_, respectively, in chondrocytes induced by IL-1β; **B**–**C** CCK-8 (**B**) and EdU (**C**) assays of the proliferation of IL-1β-induced chondrocytes; **D** ELISA determination of the levels of inflammatory factors TNF-α, IL-6, and IL-10; **E** flow cytometry measurement of chondrocyte apoptosis; **F** western blot analysis of the expression of apoptotic proteins Bax, Bcl-2, and cleaved caspase 3 in chondrocytes induced by IL-1β. Data were expressed as mean ± standard deviation, and each experiment was repeated 3 times. *P < 0.05, compared with the oe-NC + sh-NC group; ^#^*P* < 0.05, compared with the oe-Gm37494 + sh-NC group

The above experiments demonstrated that miR-181a-5p targeted and regulated GABRA1. To define whether Gm37494 alleviated chondrocyte damage induced by IL-1β through down-regulating miR-181a-5p to alter GABRA1 expression, oe-Gm37494 vector and sh-GABRA1 or sh-NC vector were simultaneously transfected into IL-1β-treated chondrocytes. Specifically, qRT-PCR and western blot manifested that the oe-Gm37494 + sh-NC group had the notably elevated GABRA1 mRNA and GABA_A_R_α1_ protein levels compared with the oe-NC + sh-NC group (*P < 0.05), which was contrary to the oe-Gm37494 + sh-GABRA1 group versus the oe-Gm37494 + sh-NC group (Fig. 6A, ^#^*P* < 0.05). Furthermore, chondrocyte damage was assessed. As for CCK-8 and EdU assays, there existed noticeable reductions of chondrocyte proliferation in the oe-Gm37494 + sh-GABRA1 group relative to the oe-Gm37494 + sh-NC group (Fig. 6B, C, ^#^*P* < 0.05). In addition, ELISA data displayed observably down-regulated IL-10 levels and dramatically up-regulated TNF-α and IL-6 levels in in the oe-Gm37494 + sh-GABRA1 group in contrast to the oe-Gm37494 + sh-NC group (Fig. 6D, ^#^*P* < 0.05). It was observed in the results of flow cytometry and western blot that chondrocyte apoptosis and Bax and cleaved caspase 3 levels increased but Bcl-2 levels reduced in the oe-Gm37494 + sh-GABRA1 group in comparison with the oe-Gm37494 + sh-NC group (Fig. 6E, F, ^#^*P* < 0.05). These data indicated that overexpressing Gm37494 inhibited chondrocyte damage induced by IL-1β via GABRA1 up-regulation.

## Discussion

OA is a prevalent joint disease with progressive pain, and the pathogeny of the disease remains unclear [25]. In recent years, the research on OA pathogenesis and treatment has focused on molecular mechanisms involving lncRNAs and miRs [26, 27]. This study was designed to gain novel insight into the mechanism of Gm37494 in OA progression with the involvement of miR-181a-5p and GABRA1. The data obtained by this study elucidated that Gm37494 overexpression ameliorated OA-induced chondrocyte damage by down-regulating miR-181a-5p and up-regulating GABRA1.

LncRNAs have been documented to participate in OA progression due to their abnormal expression [28]. For instance, lncRNA HOTAIR was proven to be implicated in the regulation of IL-1β-induced matrix metalloproteinase up-regulation and promote chondrocyte apoptosis in temporomandibular joint OA [27]. In addition, lncRNA KLF3-AS1 up-regulation was involved in the alleviatory effects of human mesenchymal stem cell-derived exosomes on OA-induced chondrocyte damage [24]. Furthermore, Huang et al. observed the poor expression of lncRNA DILC in the plasma of OA patients [29]. Gm37494 is a rarely investigated lncRNA, with only the study of Shao et al. reporting its suppressive effects on inflammation in spinal cord injury by diminishing the levels of the inflammatory factors TNF-α, IL-1β, and IL-6 [10]. Because of the critical implication of inflammation in OA development [30, 31], it is of great interests to ascertain the function of Gm37494 in OA. Intriguingly, our study found that Gm37494 expression was poor in the OA mouse model and IL-1β-treated chondrocytes. Our further cell experiments uncovered that up-regulating Gm37494 repressed apoptosis and facilitated proliferation in IL-1β-treated chondrocytes. More importantly, partially similar to the data of Shao et al., our data disclosed that overexpression of Gm37494 relieved IL-1β-induced chondrocyte inflammation by down-regulating TNF-α and IL-6 and up-regulating IL-10. In conclusion, Gm37494 was supposed to play a repressive role in OA progression.

It is extensively recognized that lncRNAs modulate the pathophysiological processes of numerous diseases including OA through the lncRNA-miR-mRNA network [32, 33]. For example, Gm37494 mitigated SCI through PPARγ by binding to miR-130b-3p [10]. Therefore, the Gm37494-miR-mRNA network in OA was identified in this study. Here, our research firstly elaborated that miR-181a-5p bound to and was inversely mediated by Gm37494 in IL-1β-treated chondrocytes. A growing body of evidence revealed that miR-181a acted as a mediator of cartilage degeneration and participated in the promotion of inflammation and death in chondrocytes [18, 34]. Another study also reported that miR-181a-5p-down-regulated growth arrest-specific gene 1 might be a mechanism of OA development [26]. More importantly, prior research elucidated that miR-181a-5p expression was high in IL-1β-treated C28/I2 cells and that ectopic miR-181a-5p neutralized the repressive impacts of circ_0020093 up-regulation on the inflammation and apoptosis of IL-1β-induced C28/I2 cells [35]. Herein, corroborating findings were tested in our research that miR-181a-5p expression was up-regulated in OA mice and the cartilage tissues of OA patients, which illustrated that miR-181a-5p was a pivotal molecule in OA. It was speculated that Gm37494 might alleviate chondrocyte damage induced by IL-1β via miR-181a-5p down-regulation. Interestingly, lncRNA small nucleolar RNA host gene 5-inhibited miR-181a-5p resulted in chondrocyte proliferation and reduced apoptosis and inflammation under the condition of IL-1β-induced OA [36]. Likewise, evidence unveiled that lncRNA nuclear enriched abundant transcript 1 could trigger miR-181a down-regulation to suppress apoptosis and inflammation in OA chondrocytes [37]. Partially concordant with these observations, our data unraveled that miR-181a-5p overexpression annulled the ameliorating impacts of Gm37494 up-regulation on IL-1β-induced chondrocyte damage and inflammation.

To further elucidate the Gm37494-miR-mRNA network, a target gene of miR-181a-5p was explored. In our study, the direct targeting relationship between miR-181a-5p and GABRA1 was observed by dual-luciferase reporter gene and RNA pull-down assays. Of note, the anti-inflammatory effects of propofol on Th2-type asthma inflammation were achieved by causing apoptosis during Th2 cell differentiation via the activation of the GABA receptor [38]. GABA also played an inhibitory role in subcutaneous adipose inflammation in obesity, and pharmacological modulation of the GABA receptor affected the inflammation in inguinal adipose tissues [39]. Nevertheless, no research reported the function of GABRA1 in OA. Therefore, it is necessary to clarify the effect of GABRA1 on OA based on the role of GABA receptor in inflammation and the binding of miR-181a-5p and GABRA1. In this context, GABRA1 mRNA and GABA_A_R_α1_ protein levels were examined in OA mice and the cartilage tissues of OA patients, which manifested decreased GABRA1 mRNA and GABA_A_R_α1_ protein levels. Of note, small interfering RNA [siRNA, one form of RNA interference (RNAi)]-induced gene dysregulation participates in the mediation of musculoskeletal conditions, such as tendon homeostasis and rheumatoid arthritis [40, 41]. Like siRNAs, shRNAs, another form of RNAi, may be transfected as plasmid vectors encoding shRNAs transcribed by RNA pol III or modified pol II promoters [42]. Therefore, a rescue experiment using overexpression vectors of Gm37494 and shRNA vectors of GABRA1 was further performed to ascertain the role of GABRA1 in IL-1β-induced chondrocyte damage, which elucidated that Gm37494 overexpression distinctly accelerated IL-1β-treated chondrocyte proliferation and constrained their apoptosis and inflammation, which was abolished by additional GABRA1 silencing. The finding indicated that overexpressing Gm37494 curtailed IL-1β-induced chondrocyte damage and inflammation by enhancing GABRA1 expression in accord with our hypothesis.

## Conclusion

In conclusion, Gm37494 and GABRA1 were down-regulated in OA mice with up-regulated miR-181a-5p. Moreover, Gm37494 prevented OA-induced chondrocyte damage by restraining the inhibition of miR-181a-5p on GABRA1. This study elucidated the functional role of Gm37494 in OA and further explored the regulation of miR-181a-5p and GABRA1. Our data provide new insight into the mechanisms of chondrocyte damage in OA and Gm37494 may become a promising treatment target for OA.

## Supplementary Information

**Additional file 1.** Mechanism diagram: In OA models, lncRNA Gm37494 upregulated GABAR1 expression by binding to miR-181a-5p, thereby reducing tissue damage caused by OA.

## Data Availability

The datasets used or analyzed during the current study are available from the corresponding author on reasonable request.

## Abbreviations

LncRNA: Long non-coding RNA
OA: Osteoarthritis
miR: MicroRNA
OARSI: Osteoarthritis Research Society International
FBS: Fetal bovine serum
CCK-8: Cell Counting Kit-8
EdU: 5-Ethynyl-2′-deoxyuridine
ELISA: Enzyme-linked immunosorbent assay
TNF-α: Tumor necrosis factor-α
IgG: Immunoglobulin G
qRT-PCR: Quantitative real-time polymerase chain reaction
NC: Negative control
RIP: RNA binding protein immunoprecipitation
